# Impact of radiation doses on clinical relapse of biochemically recurrent prostate cancer after prostatectomy

**DOI:** 10.1038/s41598-023-50434-4

**Published:** 2024-01-02

**Authors:** Seiya Takano, Natsuo Tomita, Masanari Niwa, Akira Torii, Taiki Takaoka, Nozomi Kita, Kaoru Uchiyama, Mikiko Nakanishi-Imai, Shiho Ayakawa, Masato Iida, Yusuke Tsuzuki, Shinya Otsuka, Yoshihiko Manabe, Kento Nomura, Yasutaka Ogawa, Akifumi Miyakawa, Akihiko Miyamoto, Shinya Takemoto, Takahiro Yasui, Akio Hiwatashi

**Affiliations:** 1 Department of Radiology, Nagoya City University Graduate School of Medical Sciences, 1 Kawasumi, Mizuho-Cho, Mizuho-Ku, Nagoya, Aichi 467-8601 Japan; 2 Department of Radiology, Kariya Toyota General Hospital, 5-15 Sumiyoshi-Cho, Kariya, Aichi 448-8505 Japan; 3 Department of Radiology, Japanese Red Cross Aichi Medical Center Nagoya Daini Hospital, 2-9 Myoken-Cho, Showa-Ku, Nagoya, Aichi 466-8650 Japan; 4 Department of Radiology, Japan Community Health Care Organization Chukyo Hospital, 1-1-10 Sanjo, Minami-Ku, Nagoya, Aichi 457-8510 Japan; 5 Department of Radiation Oncology, Suzuka General Hospital, 1275-53 Yamanoue, Yasuzuka-Cho, Suzuka, Mie 513-0818 Japan; 6 Department of Radiation Oncology, Nagoya Proton Therapy Center, Nagoya City West Medical Center, 1-1-1 Hirate-Cho, Kita-Ku, Nagoya, Aichi 462-8508 Japan; 7 Department of Radiology, Okazaki City Hospital, 3-1 Goshoai, Koryuji-Cho, Okazaki, Aichi 444-8553 Japan; 8 Department of Radiation Oncology, Nanbu Tokushukai General Hospital, 171-1 Hokama, Yaese-Cho, Shimajiri, Okinawa 901-0493 Japan; 9 Department of Radiotherapy, Nagoya City West Medical Center, 1-1-1 Hirate-Cho, Kita-Ku, Nagoya, Aichi 462-8508 Japan; 10 Department of Radiation Oncology, Kasugai Municipal Hospital, 1-1-1 Takaki-Cho, Kasugai, Aichi 486-8510 Japan; 11 Department of Radiation Oncology, National Hospital Organization Nagoya Medical Center, 4-1-1, Sannomaru, Naka-Ku, Nagoya, Aichi 460-0001 Japan; 12 Department of Radiation Oncology, Hokuto Hospital, 7-5 Kisen, Inada-Cho, Obihiro, Hokkaido 080-0833 Japan; 13 Department of Radiation Oncology, Fujieda Heisei Memorial Hospital, 123-1 Mizukami-Cho, Fujieda, Shizuoka 426-8662 Japan; 14 Department of Urology, Nagoya City University Graduate School of Medical Sciences, 1 Kawasumi, Mizuho-Cho, Mizuho-Ku, Nagoya, Aichi 467-8601 Japan

**Keywords:** Prostate cancer, Radiotherapy

## Abstract

The relationship between radiation doses and clinical relapse in patients receiving salvage radiotherapy (SRT) for biochemical recurrence (BCR) after radical prostatectomy (RP) remains unclear. We identified 292 eligible patients treated with SRT between 2005 and 2018 at 15 institutions. Clinical relapse-free survival (cRFS) between the ≥ 66 Gy (n = 226) and < 66 Gy groups (n = 66) were compared using the Log-rank test, followed by univariate and multivariate analyses and a subgroup analysis. After a median follow-up of 73 months, 6-year biochemical relapse-free survival, cRFS, cancer-specific survival, and overall survival rates were 58, 92, 98, and 94%, respectively. Six-year cRFS rates in the ≥ 66 Gy and < 66 Gy groups were 94 and 87%, respectively (*p* = 0.022). The multivariate analysis revealed that Gleason score ≥ 8, seminal vesicle involvement, PSA at BCR after RP ≥ 0.5 ng/ml, and a dose < 66 Gy correlated with clinical relapse (*p* = 0.015, 0.012, 0.024, and 0.0018, respectively). The subgroup analysis showed the consistent benefit of a dose ≥ 66 Gy in patients across most subgroups. Doses ≥ 66 Gy were found to significantly, albeit borderline, increase the risk of late grade ≥ 2 GU toxicity compared to doses < 66 Gy (14% vs. 3.2%, *p* = 0.055). This large multi-institutional retrospective study demonstrated that a higher SRT dose (≥ 66 Gy) resulted in superior cRFS.

## Introduction

Following radical prostatectomy (RP) for localized prostate cancer, approximately 30% of patients develop biochemical recurrence (BCR) within 10 years^1^. In the case of BCR after RP, salvage radiotherapy (SRT) to the prostate bed is the only curative treatment. Due to recent advances in RT techniques, including intensity-modulated radiation therapy (IMRT) and image-guided radiation therapy (IGRT), escalated doses may be delivered with reduced gastrointestinal and genitourinary late toxicities^2,3^. Several systematic reviews and meta-analyses indicated that a dose escalation in SRT in the range of 60–70 Gy improved biochemical control^4,5^. Therefore, the American Society for Radiation Oncology (ASTRO)/American Urological Association (AUA) guidelines suggested 64 Gy or slightly higher as the minimum dose to be delivered for biochemical control in SRT^6^, and 64–72 Gy in a standard fraction is currently recommended in the 2023 National Comprehensive Cancer Network (NCCN) guidelines^7^. However, the effects of a high dose on late toxicity need to be considered^8^. Although biopsy-proven gross recurrence may require higher doses according to the NCCN guidelines^7^, gross tumors are generally not detected on various diagnostic imaging techniques in early SRT with PSA < 0.5 ng/ml^9,10^. Positron emission tomography (PET) imaging with ^68^gallium-labeled prostate-specific membrane antigen ligands (^68^Ga-PSMA) may be promising for the detection of recurrent tumors^11^; however, ^68^Ga-PSMA PET is utilized only in some countries and may be difficult to detect minimal tumor burden in early SRT. In the setting of SRT, randomized control trial (RCT) has not yet proven the effects of radiation doses on clinical relapse identified on radiological imaging and/or biopsy. Therefore, in the present large multi-institutional study with a long-term follow-up, we investigated the relationship between radiation doses and clinical relapse-free survival (cRFS) in patients receiving SRT for BCR after RP.

## Methods

### Study population

**Figure 1:**
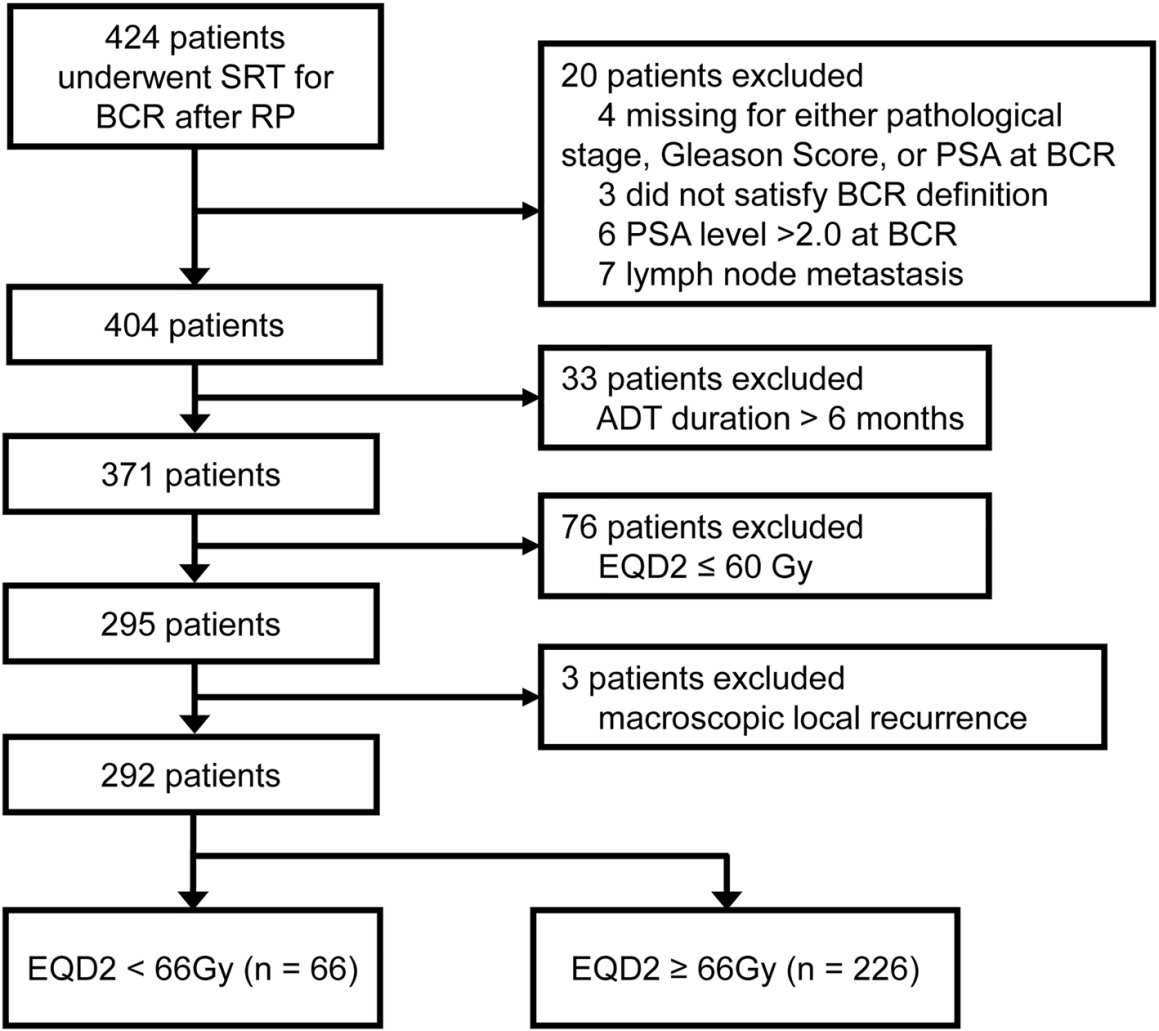
Flowchart of the study cohort. *SRT* salvage radiotherapy, *BCR* biochemical recurrence, *RP* radical prostatectomy, *PSA* prostate-specific antigen, *ADT* androgen deprivation therapy, *EQD2* equivalent dose in 2-Gy fractions.

We identified 424 patients treated with SRT for BCR after RP between 2005 and 2018 at 15 institutions. All patients had adenocarcinoma of the prostate without evidence of lymph node or distant metastasis at RP. BCR included either prostate-specific antigen (PSA) elevation or persistence after RP: PSA elevation was defined as a PSA increase ≥ 0.10 ng/ml within two or more evaluations^12^, and PSA persistence as a serum concentration ≥ 0.10 ng/ml one month after RP^13^. At least CT was performed for the evaluation of local recurrence, lymph node metastasis, and distant metastasis. Magnetic resonance imaging (MRI), bone scintigraphy, and local biopsy were not performed routinely for the evaluation of macroscopic disease. Figure 1 shows a flowchart for patient selection. Patients with missing pathological or clinical information were excluded from the analysis (n = 4). Patients receiving SRT after RP without satisfying the definition of BCR were excluded (n = 3). Patients with PSA at SRT > 2.0 ng/ml (n = 6) or with lymph node metastasis at the final pathology (n = 7) were also excluded due to the increased risk of metastases^14^. Furthermore, patients receiving SRT combined with long-term (i.e. > 6 months) androgen deprivation therapy (ADT) were excluded (n = 33) because the combination of long-term ADT may influence clinical outcomes^15^. In addition, patients treated with an equivalent dose in 2-Gy fractions (EQD2) of ≤ 60 Gy were excluded (n = 76) because a total dose of ≤ 60 Gy in conventional fractionation was insufficient for local control^16^. Patients with macroscopic disease at SRT were also excluded (n = 3). The three patients with macroscopic disease had a tumor only around the prostate bed. These exclusions yielded 292 patients in the study cohort. To assess the relationship between radiation doses and clinical outcomes, the cohort was further divided into the < 66 Gy (n = 66) and ≥ 66 Gy groups (n = 226). This division was based on the median total dose of 66 Gy for the entire patient population (n = 295) as the cutoff and was consistent with findings from previous retrospective series^17,18^. In the present study, all doses were expressed in EQD2 calculated for prostate cancer (α/β = 1.5 Gy). The present study was conducted in accordance with the 1964 Declaration of Helsinki and its later amendments. This study was approved by each institutional review board of Nagoya City University Graduate School of Medical Sciences, Kariya Toyota General Hospital, Japanese Red Cross Aichi Medical Center Nagoya Daini Hospital, Japan Community Health care Organization Chukyo Hospital, Suzuka General Hospital, Konan Kosei Hospital, Okazaki City Hospital, Nanbu Tokushukai General Hospital, Nagoya City West Medical Center, Narita Memorial Hospital, Kasugai Municipal Hospital, National Hospital Organization Nagoya Medical Center, Hokuto Hospital, Jisenkai Aizawa Hospital, and Fujieda Heisei Memorial Hospital. Since this was a retrospective observational analysis, the Nagoya City University Ethics Committee waived the need for informed consent as part of the study approval in line with the Ethical Guidelines for Medical and Health Research Involving Human Subjects in Japan. Therefore, research content was disclosed in the form of opt-out on the website.

### Treatment procedures

SRT was delivered to the prostate and seminal vesicle bed at a median (range) dose of 66 Gy (61–85). All patients were treated using 6–18-MV photon beams. Conventional fractionation (1.8–2.0 Gy per fraction) was used for 220 (75%) patients, while moderate hypo-fractionation (2.1–3.0 Gy per fraction) was used for 72 (25%) patients. Whole-pelvic radiotherapy (WPRT) was administered to 11 patients (4%) at a median (range) dose of 45 Gy (40–54) at the discretion of radiation oncologists. IMRT and IGRT were used for 191 (65%) and 217 (74%) patients, respectively. Details of our RT methods were previously described^8,10^. In general, the delineation of targets adhered to the guidelines of the Australian and New Zealand Radiation Oncology Genito-Urinary Group^19^. The prescription dose and fractionation were determined at the discretion of each radiation oncologist. The use of ADT combined with SRT was at the discretion of each urologist. A luteinizing hormone-releasing hormone analog and/or anti-androgen therapy (i.e., bicalutamide) was used for 16 patients (6%) as a neoadjuvant and/or concurrent short-term ADT. The median (range) duration of ADT was 2.5 (1–6) months.

### Clinical outcomes and toxicities

The follow-up time was calculated from the start date of SRT. The primary endpoint was cRFS, defined as the time from the start date of SRT to clinical relapse including local recurrence in the prostate bed, retroperitoneal lymph node metastasis, skeletal metastasis, and visceral metastasis. Metastases were identified on radiological imaging and/or eventual biopsy. Secondary endpoints included biochemical relapse-free survival (bRFS), cancer-specific survival (CSS), and overall survival (OS). Biochemical RFS, CSS, and OS were defined as the time from the start date of SRT to two consecutive PSA values ≥ 0.20 ng/ml, death or complications from prostate cancer, and death from any cause, respectively.

Genitourinary (GU) and gastrointestinal (GI) toxicities following SRT were assessed according to National Cancer Institute Common Terminology Criteria for Adverse Events version 4.0. Any symptoms related to GU or GI toxicities that occurred or persisted three months after the end of SRT were regarded as late toxicities. Toxicity assessments were conducted at each follow-up visit.

### Statistical analysis

Patient and treatment characteristics were compared using Fisher’s exact test for categorical variables and the Mann–Whitney *U* test for continuous variables between the < 66 Gy and ≥ 66 Gy groups. Univariate and multivariate analyses were conducted with Cox’s proportional hazards models to identify independent risk factors related to clinical relapse. Variables for the multivariate analysis were selected based on their biological importance and alignment with the predictive factors employed in the previous literature^14^. Survival was estimated with the Kaplan–Meier method, and survival estimates were compared using the Log-rank test between the two dose groups. We conducted a subgroup analysis by prognostic factors identified in the multivariate analysis. To address the potential bias due to differences in patient characteristics between the two groups, we conducted sensitivity analyses comparing survival curves of cRFS by excluding cases with short-term ADT administration or stratifying by the start year of SRT. The start year of SRT was divided at the median value of 2014 (Table 1). Late grade 2 or higher GU and GI toxicities were analyzed by estimating cumulative incidence curves, treating death from any cause as a competing risk. Gray’s test stratified by total doses in EQD2 was performed for late grade 2 or higher GU and GI toxicities. All statistical analyses were conducted using EZR^20^, which is a graphical user interface for R (version 3.6.3; R Foundation for Statistical Computing, Vienna, Austria). The threshold for significance was *p* < 0.05.

**Table 1:** Patient and treatment characteristics.

Characteristic	All patients	< 66 Gy group	≥ 66 Gy group	*p*-value
Total	n = 292	n = 66	n = 226	
Age at SRT (year)	68 (49–80)	70 (52–77)	68 (49–80)	0.22
Gleason score				0.013
≤ 6	30 (10%)	11 (17%)	19 (8.4%)	
7	161 (55%)	41 (62%)	120 (53%)	
8–10	101 (35%)	14 (21%)	87 (39%)	
T stage				0.68
≤ pT2c	159 (55%)	38 (58%)	121 (54%)	
pT3a	88 (30%)	17 (26%)	71 (31%)	
pT3b	45 (15%)	11 (17%)	34 (15%)	
ECE +	123 (42%)	23 (35%)	100 (44%)	0.20
SVI +	45 (15%)	11 (17%)	34 (15%)	0.70
Surgical margin +	172 (59%)	33 (50%)	139 (62%)	0.12
Preoperative initial PSA (ng/ml)	11.4 (4.00–84.1)	10.8 (4.00–56.9)	11.7 (4.18–84.1)	0.59
Postoperative PSA nadir (ng/ml)	0.04 (0.00–1.82)	0.06 (0.00–1.74)	0.04 (0.00–1.82)	0.83
PSA at BCR after RP (ng/ml)	0.38 (0.10–1.94)	0.32 (0.10–1.94)	0.42 (0.10–2.00)	0.012
EQD2 (Gy)	66.0 (61.0–85.0)	64.0 (61.0–65.0)	67.0 (66.0–85.0)	< 0.001
WPRT	11 (4%)	2 (3%)	9 (4%)	1.0
Short-term ADT use with SRT	16 (6%)	2 (3%)	14 (6%)	0.54
Follow-up time (month)^a^	73 (5–189)	98 (11–166)	68 (5–189)	0.001
2006–2013	n = 131	n = 54	n = 77	
	102 (6–189)	114 (11–166)	100 (6–189)	0.25
2014–2018	n = 161	n = 12	n = 149	
	60 (5–97)	55 (23–92)	61 (5–97)	0.42
Total	n = 118	n = 23	n = 95	
PSA at BCR after SRT (ng/ml)	0.30 (0.01–4.32)	0.35 (0.21–3.21)	0.30 (0.01–4.32)	0.24
ADT use in patients with recurrence after SRT	99 (84%)	21 (91%)	78 (82%)	0.36

Data are n (%) or medians (range).

*SRT* salvage radiotherapy, *ECE* extracapsular extension, *SVI* seminal vesicle involvement, *PSA* prostate-specific antigen, *BCR* biochemical recurrence, *RP* radical prostatectomy, *EQD2* equivalent dose in 2-Gy fractions, *WPRT* whole-pelvic radiotherapy, *ADT* androgen deprivation therapy.

^a^The follow-up time was stratified by the year of the initiation of SRT: 2006–2013 and 2014–2018.

## Results

### Patient characteristics

Table 1 shows patient characteristics. The median dose for all patients was 66.0 Gy (61.0–85.0) in EQD2. Gleason scores and PSA at BCR after RP were significantly higher in the ≥ 66 Gy group. Follow-up times were significantly longer in the < 66 Gy group (*p* = 0.001, Table 1); however, when stratified by the start year of SRT (2006–2013 and 2014–2018), there were no significant differences in follow-up time between the < 66 Gy and ≥ 66 Gy groups (*p* = 0.25 and 0.42, respectively, Table 1). Among patients with BCR after SRT (n = 118), 99 (84%) subsequently received ADT. Between the two dose groups, no significant differences were found in the other characteristics (Table 1).

### Outcomes and impact of radiation doses

**Figure 2:**
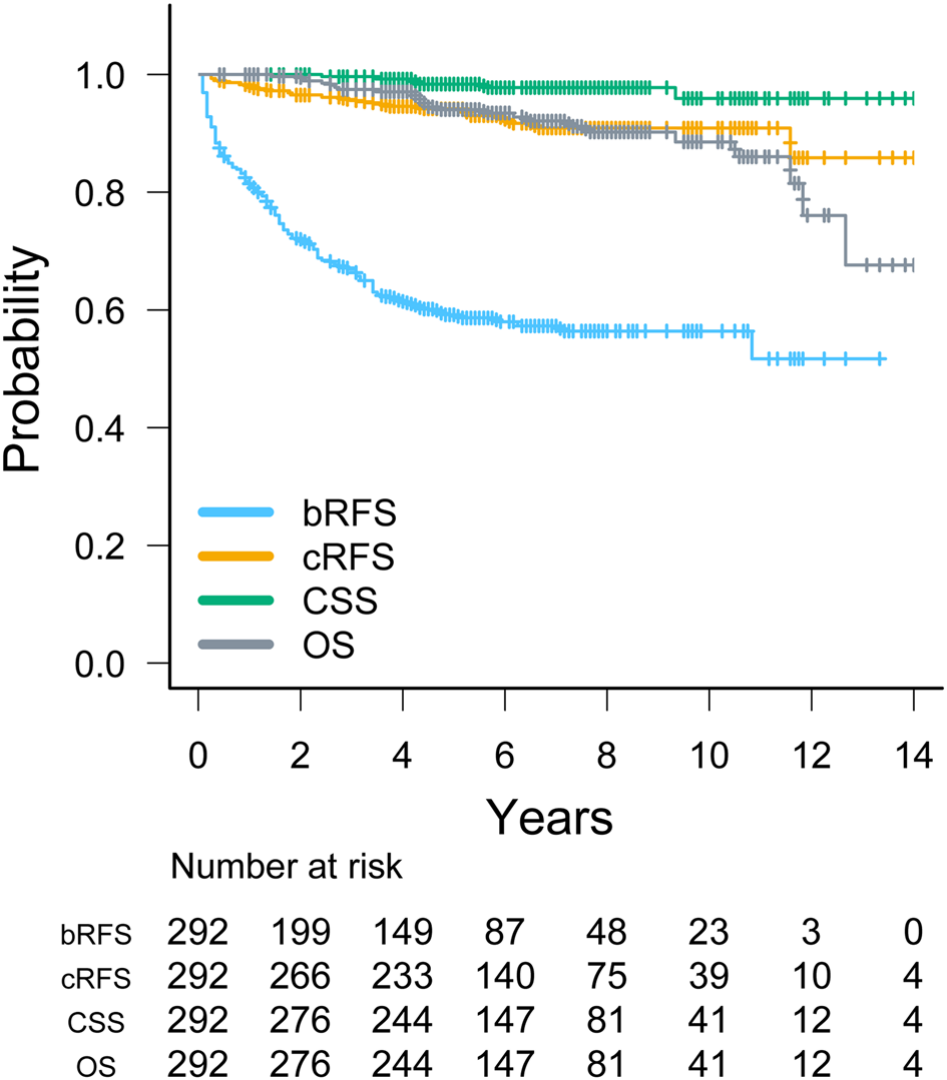
Kaplan–Meier curves of biochemical relapse-free survival (bRFS), clinical relapse-free survival (cRFS), cancer-specific survival (CSS), and overall survival (OS) for all patients (n = 292) receiving salvage radiotherapy for biochemical recurrence after radical prostatectomy.

The median follow-up duration was 73 months (range, 5–189) for all patients (n = 292). Among all patients, 25 (8%) died, 6 (2%) of whom died of prostate cancer. In 118 patients (40%) with BCR after SRT, 22 (8%) developed clinical relapse. The median PSA at BCR after SRT was 0.30 (0.01–4.32) ng/ml. Figure 2 shows the survival curves of bRFS, cRFS, CSS, and OS for all patients receiving SRT for BCR after RP. Six-year bRFS, cRFS, CSS, and OS rates were 58% (95% confidence interval [CI], 52–64), 92% (95% CI, 88–95), 98% (95% CI, 95–99), and 94% (95% CI, 90–96), respectively.

**Figure 3:**
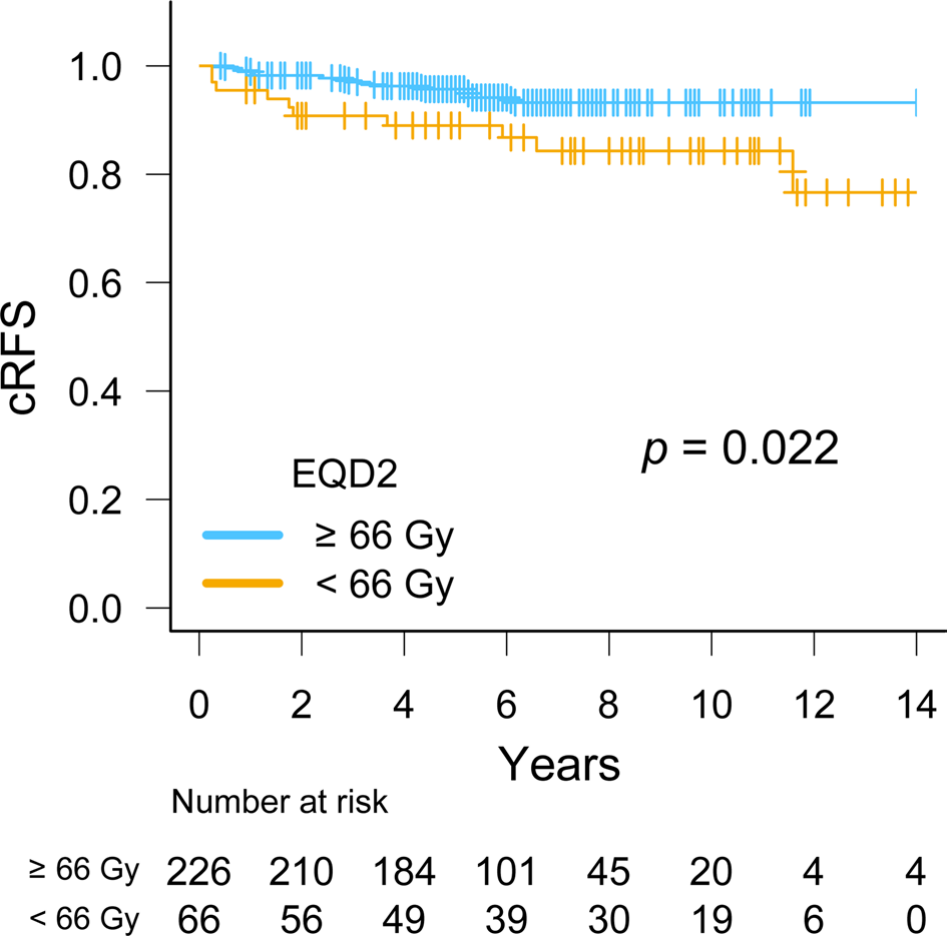
Kaplan–Meier curves of clinical relapse-free survival (cRFS) for patients receiving total doses of ≥ 66 Gy (n = 226) vs. < 66 Gy (n = 66) in an equivalent dose in 2-Gy fractions (EQD2).

Twenty-two patients (8%) developed clinical relapse: 3 with regional recurrence, 15 with distant metastases, and 4 with both regional recurrence and distant metastases. The sites of distant metastases were bone in 15 patients, the lungs in 3, the liver in 3, and extraregional lymph nodes in 3. No patients developed local recurrence. The median (range) times to clinical relapse from the start date of SRT were 32 months (3–139) for all patients, 36 months (6–73) for the ≥ 66 Gy group, and 22 months (3–139) for the < 66 Gy group. Table 2 summarizes comparisons of biochemical and clinical relapse, prostate cancer death, and overall death between the two dose groups. Doses ≥ 66 Gy significantly improved cRFS (*p* = 0.022, Fig. 3); the 6-year cRFS rates of the ≥ 66 Gy and < 66 Gy groups were 94% (95% CI, 90–97) and 87% (95% CI, 75–93), respectively (*p* = 0.022, Table 2). Excluding patients who received short-term ADT (n = 16) yielded similar results regarding the effect of doses ≥ 66 Gy on cRFS (Supplementary Fig. S1a, *p* = 0.031). In the 2006–2013 period, there was still a significant improvement in cRFS with doses ≥ 66 Gy (Supplementary Fig. S1b, *p* = 0.040). In the 2014–2018 period, a similar trend was observed (Supplementary Fig. S1c), although there was no statistical significance (*p* = 0.54).

**Table 2:** Summary of clinical outcomes for patients receiving total doses of ≥ 66 Gy vs. < 66 Gy in an Equivalent dose in 2-Gy fractions.

Dose group	Event	6y-bRFS	*p*-value	Event	6y-cRFS	*p*-value	Event	6y-CSS	*p*-value	Event	6y-OS	*p*-value
≥ 66 Gy (n = 226)	95 (42%)	56 (49–62)	0.21	12 (5%)	94 (90–97)	0.022	0	100	< 0.001	11 (5%)	96 (92–98)	0.006
< 66 Gy (n = 66)	23 (35%)	66 (52–76)		10 (15%)	87 (75–93)		6 (9%)	91 (79–96)		14 (21%)	86 (74–93)	
Total (n = 292)	118 (40%)	58 (52–64)		22 (8%)	92 (88–95)		6 (2%)	98 (95–99)		25 (8%)	94 (90–96)	

Data are n (%) or % (95% confidence interval).

*bRFS* biochemical relapse-free survival, *cRFS* clinical relapse-free survival, *CSS* cancer-specific survival, *OS* overall survival.

In the study cohort, 118 patients (40%) developed BCR after SRT. The 6-year bRFS rates of the ≥ 66 Gy and < 66 Gy groups were 56% (95% CI, 49–62) and 66% (95% CI, 52–76), respectively (*p* = 0.21, Table 2). The 6-year CSS rates of the ≥ 66 Gy and < 66 Gy groups were 100% (95% CI, not estimable) and 91% (95% CI, 79–96), respectively (*p* < 0.001, Table 2). The 6-year OS rates of the ≥ 66 Gy and < 66 Gy groups were 96% (95% CI, 92–98) and 86% (95% CI, 74–93), respectively (*p* = 0.006, Table 2).

### Prognostic factors for clinical relapse-free survival and a subgroup analysis

**Figure 4:**
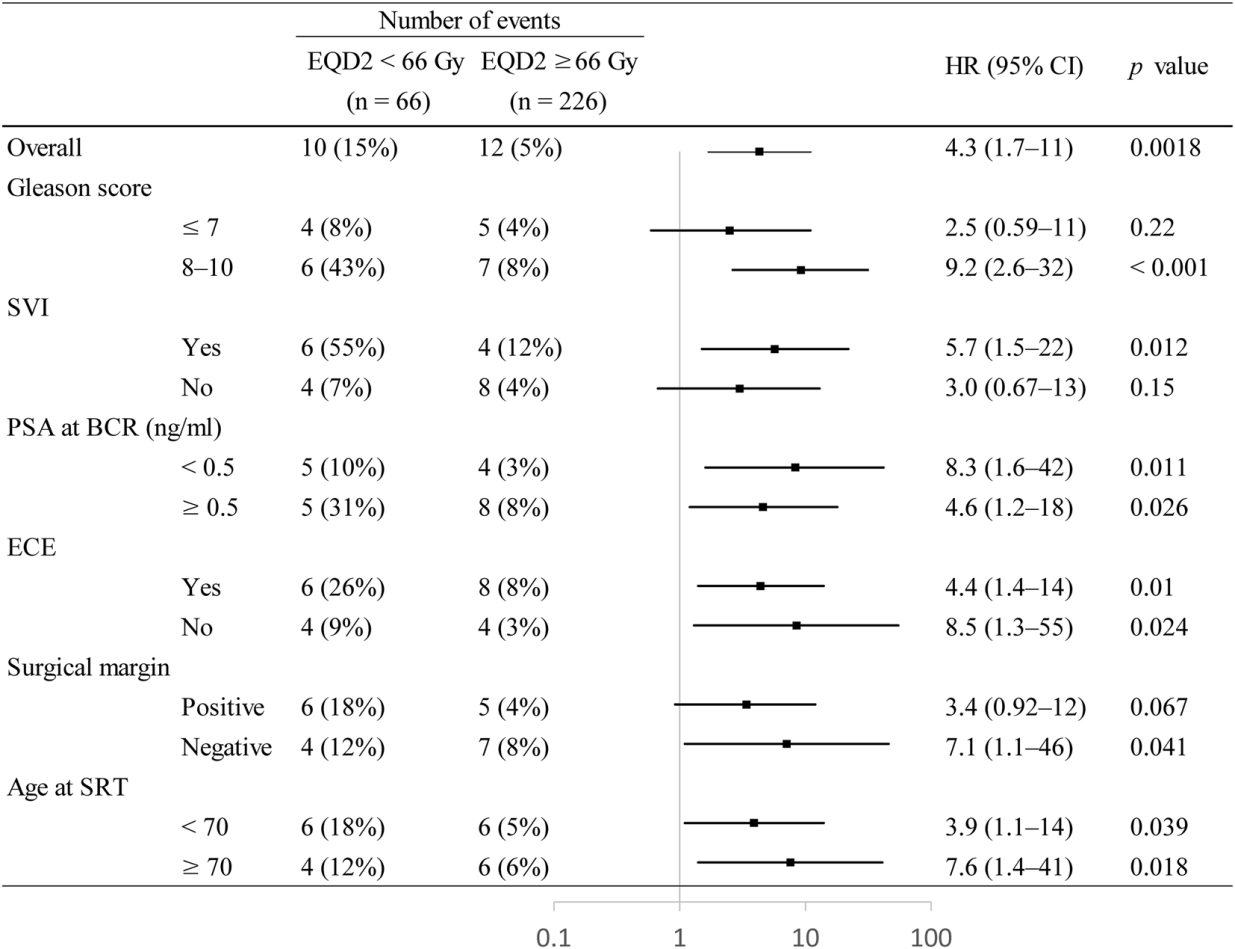
Effects of radiation doses by clinical and pathological risk factors. *EQD2* equivalent dose in 2-Gy fractions, *HR* hazard ratio, *95% CI* 95% confidence interval, *SVI* seminal vesicle involvement, *PSA* prostate-specific antigen, *BCR* biochemical recurrence, *RP* radical prostatectomy, *ECE* extracapsular extension, *SRT* salvage radiotherapy.

Table 3 shows the results of univariate and multivariate analyses for clinical relapse after SRT. Gleason score ≥ 8, SVI, PSA at BCR after RP ≥ 0.5 ng/ml, and EQD2 < 66 Gy correlated with clinical relapse in the univariate and multivariate analyses. Figure 4 shows the number of events and adjusted hazard ratios (HRs) of clinical relapse by prognostic factors in the subgroup analysis. The most prominent benefit with a dose of ≥ 66 Gy was observed in patients with Gleason scores 8–10 (HR, 9.2; 95% CI, 2.6–32; *p* < 0.001). This benefit was consistent in patients across the majority of subgroups, including PSA at BCR after RP, ECE, and age at SRT. Multivariate analyses of CSS and OS were not performed because of the small number of cancer-specific deaths (n = 6).

**Table 3:** Univariate and multivariate analyses of clinical and pathological factors predictive of clinical relapse after salvage radiotherapy.

Predictor	Univariate		Multivariate	
	HR (95% CI)	*p*-value	HR (95% CI)	*p*-value
Age at SRT < 70	0.81 (0.35–1.89)	0.63	0.58 (0.23–1.47)	0.25
Gleason score ≥ 8	3.07 (1.30–7.25)	0.01	3.16 (1.25–7.98)	0.015
ECE +	2.40 (1.00–5.71)	0.049	1.74 (0.64–4.71)	0.28
SVI +	5.37 (2.31–12.7)	< 0.001	3.55 (1.33–9.52)	0.012
Surgical margin −	1.57 (0.68–3.63)	0.29	2.06 (0.83–5.09)	0.12
PSA at BCR after RP ≥ 0.50 ng/ml	2.51 (1.07–5.87)	0.034	2.90 (1.15–7.29)	0.024
EQD2 < 66 Gy	2.61 (1.12–6.12)	0.027	4.31 (1.72–10.81)	0.0018

*HR* hazard ratio, *95% CI* 95% confidence interval, *SRT* salvage radiotherapy, *ECE* extracapsular extension, *SVI* seminal vesicle involvement, *PSA* prostate-specific antigen, *BCR* biochemical recurrence, *RP* radical prostatectomy, *EQD2* equivalent dose in 2-Gy fractions.

### Late toxicities

**Figure 5:**
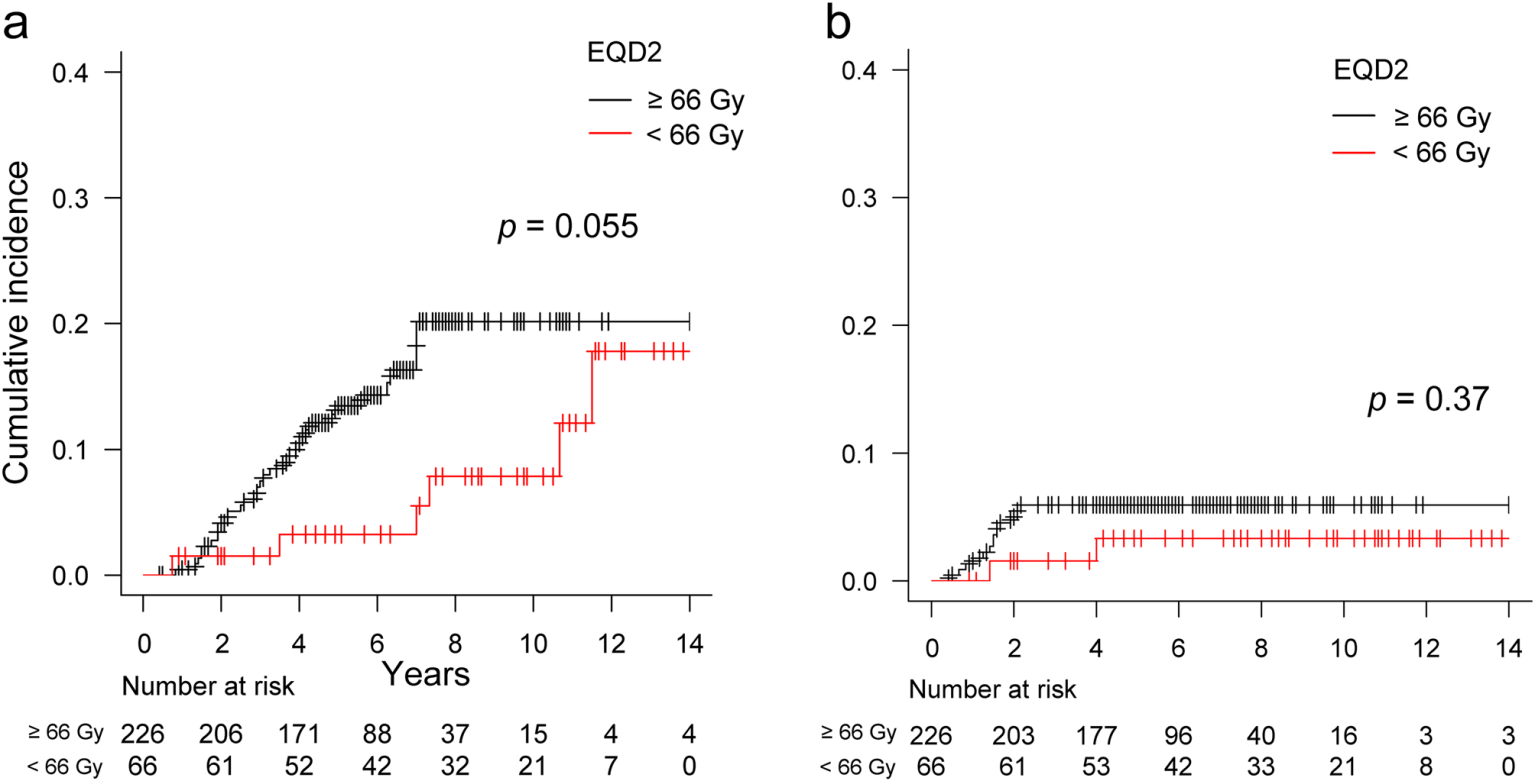
Cumulative incidence curves of late grade 2 or higher (**a**) genitourinary and (**b**) gastrointestinal toxicities for patients receiving total doses of ≥ 66 Gy (n = 226) vs. < 66 Gy (n = 66) in an equivalent dose in 2-Gy fractions (EQD2).

The symptoms of the late GU and GI toxicities are shown in Table S1. Among all patients (n = 292), late grade 1, 2, 3, 4, and 5 GU toxicities were reported in 43 (15%), 26 (8.9%), 16 (5.5%), 1 (0.3%), and 1 patient (0.3%), respectively. The most frequent symptom of late grade 2 or higher GU toxicity was hematuria in 30 patients (10%) followed by urinary tract obstruction in 8 patients (2.7%). The patient with late grade 4 GU toxicity underwent surgery for bladder tamponade resulting from hematuria. The patient with late grade 5 GU toxicity experienced postrenal acute renal failure due to urinary obstruction. The 6-year cumulative incidence of late grade 2 or higher GU toxicities for all patients was 12% (95% CI, 8.1–16). The 6-year cumulative incidence of late grade 2 or higher GU toxicities was higher in the ≥ 66 Gy group than in the < 66 Gy group with borderline significance (Fig. 5a, 14% [95% CI, 9.7–20] vs. 3.2% [95% CI, 0.6–10], *p* = 0.055).

Late grade 1, 2, and 3 GI toxicities were reported in 35 (12%), 8 (2.7%), and 8 patients (2.7%), respectively. No grade 4 or 5 GI toxicities were reported. The most frequent symptom of late grade 2 or higher GI toxicity was rectal hemorrhage in 15 patients (5.1%). The 6-year cumulative incidence of late grade 2 or higher GU toxicities for all patients was 5.4% (95% CI, 3.1–8.4). There was no significant difference in the 6-year cumulative incidence of late grade 2 or higher GI toxicities between the ≥ 66 Gy and < 66 Gy groups (Fig. 5b, 6.0% [95% CI, 3.3–9.6] vs. 3.3% [95% CI, 0.6–10], *p* = 0.37).

## Discussion

We investigated the relationship between radiation doses and cRFS in patients who received SRT for BCR after RP. The present results demonstrated significantly better cRFS with a SRT dose of ≥ 66 Gy (*p* = 0.022, Fig. 3), which supports the dose recommendations in the ASTRO/AUA guidelines (64 Gy or slightly higher) ^6^ and 2023 NCCN guidelines (64–72 Gy) ^7^ even in terms of clinical relapse. To the best of our knowledge, this is the first study to show the benefit of a higher SRT dose, which reduced clinical relapse. Furthermore, the subgroup analysis confirmed a consistent benefit with a dose of ≥ 66 Gy in patients across most subgroups.

Several observational studies^14,21,22^ and a meta-analysis^5^ on SRT reported the advantage of dose escalations for biochemical control. However, the SAKK 09/10 trial^23^ and a Chinese single-center trial ^24^ did not verify the benefit of doses ≥ 70 Gy for bRFS or cRFS. In contrast, the present study showed significantly better cRFS in the ≥ 66 Gy group (*p* = 0.022, Fig. 3). This may be partly attributed to our study population having more poor prognostic factors, i.e., Gleason scores 8–10 (35%) and a T stage ≥ pT3b (15%), than other studies^17,21,23,25^. Since higher Gleason scores correlated with BCR and distant metastasis after SRT^21,26^, previous studies with more favorable prognostic factors may have included patients who did not require dose-escalated SRT. The present subgroup analysis showed the marked benefit of doses ≥ 66 Gy in patients with Gleason scores 8–10, but not with Gleason score ≤ 7 (Fig. 4).

The difference in prescribed doses may have influenced the present results. We selected 66 Gy as a dose cut-off based on previous retrospective series^17,18^, which reported that SRT doses ≥ 66 Gy improved biochemical control. A meta-analysis by King et al.^5^ showed a sigmoidal dose–response curve with a tumor control dose 50 of approximately 66 Gy. Some of the patients in our cohort received low doses (61–64 Gy) because the recommended doses were 60–66 Gy for SRT according to the guidelines of the Australian and New Zealand Radiation Oncology Genito-Urinary Group published in 2008^19^. This may have contributed to the difference in cRFS between the two dose groups (*p* = 0.020, Fig. 3) in contrast to the findings of the SAKK 09/10 trial (70 Gy vs. 64 Gy)^23^ and Chinese trial (72 Gy vs. 66 Gy)^24^. A dose range of 60–64 Gy is considered to be insufficient for local control^16^ according to the SWOG study^27^, which showed that the 10-year risk of local failure was 9% even after adjuvant radiation therapy. A possible interpretation of these findings is that at least 64 Gy is needed to eradicate microscopic disease in the prostate bed, and also that further benefits may not be expected at doses ≥ 70 Gy. However, a recent matched-pair analysis^25^ suggested that doses ≥ 70 Gy were particularly beneficial in high-risk patients. Therefore, selection bias regarding some pathological features may have largely affected clinical outcomes. While patient characteristics exhibited imbalances in terms of short-term ADT administration, excluding patients who received short-term ADT yielded the similar result regarding the improvement in cRFS with doses ≥ 66 Gy (Supplementary Fig. S1a). The limited influence of short-term ADT in our study could be ascribed to the high rate of secondary ADT use after BCR following SRT (84%) and the relatively low rate of WPRT (4%, as shown in Table 1). In addition, the median follow-up time between the two groups differed by 30 months (Table 1, *p* = 0.001). To address the potential bias in detecting metastatic disease, the data were split into the two periods (2006–2013 and 2014–2018) with similar follow-up durations (Table 1, *p* = 0.25 and 0.42). In each period, we observed a consistent trend of cRFS improvement with dose escalation (Supplementary Figs. S1b and S1c). The increased prevalence of IMRT, which allows for high-conformity irradiation^8^ may have contributed to the higher incidence of patients receiving doses ≥ 66 Gy between 2014 and 2018. Furthermore, the difference in the total dose could have been influenced by the increasing recommended doses outlined in guidelines^6^.

In contrast to previous studies^17,18^, higher doses of ≥ 66 Gy did not significantly improve bRFS (*p* = 0.21, Table 2). Heterogeneity in pathological and clinical characteristics (Table 1) may have influenced our results. In the present study, the ≥ 66 Gy group had worse prognostic factors: Gleason scores 8–10 were detected in 39 and 21% of patients in the ≥ 66 Gy and < 66 Gy groups, respectively (*p* = 0.013, Table 1); PSA at BCR after RP were 0.42 and 0.32 ng/ml in the ≥ 66 Gy and < 66 Gy groups, respectively (*p* = 0.012, Table 1). Since a higher Gleason score and PSA at BCR are associated with a higher incidence of BCR^4,9,21,28^, this imbalance in prognostic factors may have offset the difference in bRFS between the two dose groups. These prognostic factors are also associated with distant metastasis^21,26^ and death by any cause^28^. Therefore, conversely, in the present study, improved cRFS with higher doses of ≥ 66 Gy (*p* = 0.022, Fig. 3) may be strengthened because patients in the ≥ 66 Gy group had less favorable prognostic factors, due to which the between-group difference may have been underestimated (i.e., bias towards null).

We observed clinical relapse in 22 patients (7%). Sites of recurrence were as follows: 3 with regional recurrence, 15 with distant metastases, and 4 with both regional recurrence and distant metastases, but not local recurrence. This result was partly consistent with previous findings showing that the most frequent pattern of recurrence in an 8-year follow-up was distant metastasis (21%), followed by regional (6%) and local recurrence (2.2%)^29^. The assumption that SRT achieves local control and prevents or delays metastases is supported by the favorable 6-year disease-free survival observed even in patients with high-risk factors such as GS8–10 and short PSA doubling time^14^. However, no local recurrence was observed in either dose group. This may be attributed to difficulties in detecting local recurrence compared to regional recurrence and bone metastasis. There is no consensus on monitoring local recurrence after SRT^7^. Even biopsy has limited predictive value for at least two years post-RT due to delayed tumor regression^30^. The low PSA level (median, 0.30 ng/ml) and high utilization rate of ADT (84%) at BCR after SRT may also have affected the capability of detecting local recurrence. Furthermore, the retrospective nature of the present study may have led to the infrequent use of MRI and local biopsy.

WPRT and ADT may exert effects even on subclinical distant metastasis, and recent RCTs showed improvements in clinical outcomes by the addition of WPRT^31^ and concurrent short-term ADT^32^ to SRT. However, in the present study, WPRT and concurrent short-term ADT were rarely used (≤ 6%) because evidence remained unestablished when we initiated the present study. Nevertheless, we observed a significant 7% improvement in 6-year cRFS in the ≥ 66 Gy group (Table 2, *p* = 0.022), and the pattern of recurrence exclusively involved regional recurrence and distant metastasis. Future studies need to consider emerging evidence from several RCTs^31,32^ that will facilitate the application of ADT and WPRT, and the effects of dose-escalated SRT to local lesions in well-selected patients will become clear.

In the present study, the 6-year cumulative incidence of late grade 2 or higher GU toxicities was 12%, which aligned with the findings in previous literature^9,23,33,34^. Nevertheless, the observed trend shown in Fig. 5a suggests that doses ≥ 66 Gy substantially increased the risk of grade 2 or higher GU toxicity. Given the lack of clear benefits in biochemical control with doses ≥ 70 Gy from the two RCTs^23,24^ and the previous finding that total dose ≥ 68 Gy was identified as an independent risk factor for late GU toxicity^8^, the optimal total dose may have been in the range of approximately 66–68 Gy in our study population. Further studies are warranted to explore the trade-off between tumor control and adverse effects.

The present study had several limitations. Due to its retrospective nature, a selection bias was inevitable. For example, decisions regarding prescribed doses were at the discretion of radiation oncologists, and this may have introduced undetectable confounders. However, patient characteristics in each group were almost homogenous. In the present study, sensitivity analyses were conducted to mitigate potential bias arising from differences in short-term ADT use and follow-up duration between the two groups. Moreover, imaging techniques depended on each institution’s practice; MRI and local biopsy were not routinely performed during the follow-up period. This may have reduced the sensitivity of detecting local and regional recurrence. We also did not perform ^68^Ga-PSMA PET, which has a higher diagnostic capability to detect small-volume metastases^35^. In addition, we did not perform a multivariate analysis of CSS or OS because of the small number of events. Further studies with a longer follow-up period are warranted to assess clinical outcomes.

In conclusion, this large multi-institutional retrospective study demonstrated that a higher dose (≥ 66 Gy) resulted in superior 6-year cRFS in patients receiving SRT for BCR after RP. Future prospective studies need to investigate the impact of dose-escalated SRT on cRFS in well-selected patients.

### Supplementary Information

Supplementary Information.

## Data Availability

The datasets generated and/or analyzed during the present study are not publicly available due to ethical reasons, but are available from the corresponding author upon reasonable request.
