# Supplementary material for: Impact of radiation doses on clinical relapse of biochemically recurrent prostate cancer after prostatectomy
Source: Sci Rep. 2024 Jan 2;14:113. doi: 10.1038/s41598-023-50434-4 (PMC10761985; doi:10.1038/s41598-023-50434-4)
Supplement: Supplementary file 1 — Supplementary Information. [file 41598_2023_50434_MOESM1_ESM.docx]

**Impact of radiation doses on clinical relapse of** **biochemically recurrent prostate cancer after prostatectomy**

Seiya Takano^1^, Natsuo Tomita^1*^, Masanari Niwa^1^, Akira Torii^1^, Taiki Takaoka^1^, Nozomi Kita^1^, Kaoru Uchiyama^2^, Mikiko Nakanishi-Imai^3^, Shiho Ayakawa^4^, Masato Iida^5^, Yusuke Tsuzuki^6^, Shinya Otsuka^7^, Yoshihiko Manabe^8^, Kento Nomura^9^, Yasutaka Ogawa^10^, Akifumi Miyakawa^11^, Akihiko Miyamoto^12^, Shinya Takemoto^13^, Takahiro Yasui^14^, and Akio Hiwatashi^1^

^1^ Department of Radiology, Nagoya City University Graduate School of Medical Sciences, 1 Kawasumi, Mizuho-cho, Mizuho-ku, Nagoya, Aichi, 467-8601, Japan

^2^ Department of Radiology, Kariya Toyota General Hospital, 5-15 Sumiyoshi-cho, Kariya, Aichi, 448-8505, Japan

^3^ Department of Radiology, Japanese Red Cross Aichi Medical Center Nagoya Daini Hospital, 2-9 Myoken-cho, Showa-ku, Nagoya, Aichi, 466-8650, Japan

^4^ Department of Radiology, Japan Community Health care Organization Chukyo Hospital, 1-1-10 Sanjo, Minami-ku, Nagoya, Aichi, 457-8510, Japan

^5^ Department of Radiation Oncology, Suzuka General Hospital, 1275-53 Yamanoue, Yasuzuka-cho, Suzuka, Mie, 513-0818, Japan

^6^ Department of Radiation Oncology, Nagoya Proton Therapy Center, Nagoya City West Medical Center, 1-1-1 Hirate-cho, Kita-ku, Nagoya, Aichi, 462-8508, Japan

^7^ Department of Radiology, Okazaki City Hospital, 3-1 Goshoai, Koryuji-cho, Okazaki, Aichi, 444-8553, Japan

^8^ Department of Radiation Oncology, Nanbu Tokushukai General Hospital, 171-1 Hokama, Yaese-cho, Shimajiri, Okinawa, 901-0493, Japan

^9^ Department of Radiotherapy, Nagoya City West Medical Center, 1-1-1 Hirate-cho, Kita-ku, Nagoya, Aichi, 462-8508, Japan

^10^ Department of Radiation Oncology, Kasugai Municipal Hospital, 1-1-1 Takaki-cho, Kasugai, Aichi, 486-8510, Japan

^11^ Department of Radiation Oncology, National Hospital Organization Nagoya Medical Center, 4-1-1, Sannomaru, Naka-ku, Nagoya, Aichi, 460-0001, Japan

^12^ Department of Radiation Oncology, Hokuto Hospital, 7-5 Kisen, Inada-cho, Obihiro, Hokkaido, 080-0833, Japan

^13^ Department of Radiation Oncology, Fujieda Heisei Memorial Hospital, 123-1 Mizukami-cho, Fujieda, Shizuoka, 426-8662, Japan

^14^ Department of Urology, Nagoya City University Graduate School of Medical Sciences, 1 Kawasumi, Mizuho-cho, Mizuho-ku, Nagoya, Aichi, 467-8601, Japan

**Corresponding author:** Natsuo Tomita, MD, PhD, Department of Radiology, Nagoya City University Graduate School of Medical Sciences, 1 Kawasumi, Mizuho-cho, Mizuho-ku, Nagoya, Aichi, 467-8601, Japan

Phone: (+81)52-853-8276; Fax: (+81)52-852-5244; E-mail: c051728@yahoo.co.jp

ORCID: Natsuo Tomita; http://orcid.org/0000-0003-4601-0119

**Supplementary Table S1** The symptoms of the late GU and GI toxicities

|  | **All patients (n = 292)** | | | | | **< 66 Gy group (n = 66)** | | | | | | **≥ 66 Gy group (n = 226)** | | | | | |
| --- | --- | --- | --- | --- | --- | --- | --- | --- | --- | --- | --- | --- | --- | --- | --- | --- | --- |
| **Symptoms** | **Grade 1** | **Grade 2** | **Grade 3** | **Grade 4** | **Grade 5** | | **Grade 1** | **Grade 2** | **Grade 3** | **Grade 4** | **Grade 5** | | **Grade 1** | **Grade 2** | **Grade 3** | **Grade 4** | **Grade 5** |
| **Late GU toxicity** |  |  |  |  |  | |  |  |  |  |  | |  |  |  |  |  |
| Hematuria | 34 (12) | 17 (5.8) | 12 (4.1) | 1 (0.3) | 0 (0) | | 8 (12) | 0 (0) | 4 (6.1) | 1 (1.5) | 0 (0) | | 26 (12) | 17 (7.5) | 8 (3.5) | 0 (0) | 0 (0) |
| Urinary incontinence | 7 (2.4) | 3 (1) | 1 (0.3) | - | - | | 0 (0) | 0 (0) | 0 (0) | - | - | | 7 (3.1) | 3 (1.3) | 1 (0.4) | - | - |
| Urinary tract obstruction | 0 (0) | 4 (1.4) | 3 (1) | 0 (0) | 1 (0.3) | | 0 (0) | 1 (1.5) | 2 (3) | 0 (0) | 0 (0) | | 0 (0) | 3 (1.3) | 1 (0.4) | 0 (0) | 1 (0.4) |
| Urinary frequency | 2 (0.7) | 0 (0) | 0 (0) | - | - | | 0 (0) | 0 (0) | 0 (0) | - | - | | 2 (0.9) | 0 (0) | 0 (0) | - | - |
| Non-infective cystitis | 0 (0) | 1 (0.3) | 0 (0) | 0 (0) | 0 (0) | | 0 (0) | 0 (0) | 0 (0) | 0 (0) | 0 (0) | | 0 (0) | 1 (0.4) | 0 (0) | 0 (0) | 0 (0) |
| Urinary fistula | - | 1 (0.3) | 0 (0) | 0 (0) | 0 (0) | | - | 0 (0) | 0 (0) | 0 (0) | 0 (0) | | - | 1 (0.4) | 0 (0) | 0 (0) | 0 (0) |
| Total | 43 (15) | 26 (8.9) | 16 (5.5) | 1 (0.3) | 1 (0.3) | | 8 (12) | 1 (1.5) | 6 (9.1) | 1 (1.5) | 0 (0) | | 35 (16) | 25 (11) | 10 (4.4) | 0 (0) | 1 (0.4) |
| **Late GI toxicity** |  |  |  |  |  | |  |  |  |  |  | |  |  |  |  |  |
| Rectal hemorrhage | 34 (12) | 7 (2.4) | 8 (2.7) | 0 (0) | 0 (0) | | 7 (11) | 1 (1.5) | 1 (1.5) | 0 (0) | 0 (0) | | 27 (12) | 6 (2.7) | 7 (3.1) | 0 (0) | 0 (0) |
| Rectal pain | 0 (0) | 1 (0.3) | 0 (0) | - | - | | 0 (0) | 0 (0) | 0 (0) | - | - | | 0 (0) | 1 (0.4) | 0 (0) | - | - |
| Rectal stenosis | 1 (0.3) | 0 (0) | 0 (0) | 0 (0) | 0 (0) | | 1 (1.5) | 0 (0) | 0 (0) | 0 (0) | 0 (0) | | 0 (0) | 0 (0) | 0 (0) | 0 (0) | 0 (0) |
| Total | 35 (12) | 8 (2.7) | 8 (2.7) | 0 (0) | 0 (0) | | 8 (12) | 1 (1.5) | 1 (1.5) | 0 (0) | 0 (0) | | 27 (12) | 7 (3.1) | 7 (3.1) | 0 (0) | 0 (0) |
|  |  |  |  |  |  | |  |  |  |  |  | |  |  |  |  |  |

Data are shown as n (%). *GU* Genitourinary, *GI* gastrointestinal.

**Supplementary Fig. S1**

**
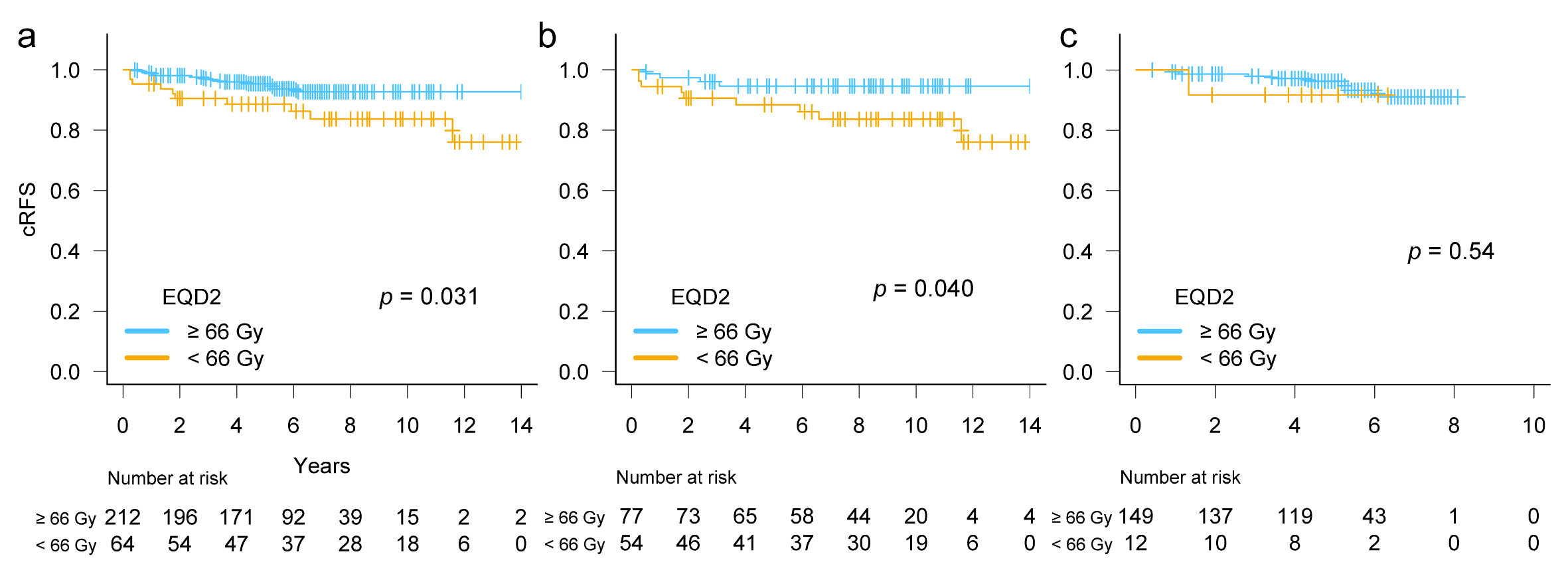
**

**Supplementary Fig. S1** Kaplan-Meier curves of clinical relapse-free survival (cRFS) for patients receiving total doses of ≥ 66 Gy (n = 226) vs. < 66 Gy (n = 66) in an equivalent dose in 2-Gy fractions (EQD2) after (a) excluding patients who received short-term androgen deprivation therapy (n = 212 in the ≥ 66 Gy group, n = 64 in the < 66 group) or stratifying by the start year of salvage radiotherapy: (b) 2006–2013 (n = 77 in the ≥ 66 Gy group, n = 54 in the < 66 group) and (c) 2014–2018 (n = 149 in the ≥ 66 Gy group, n = 12 in the < 66 group).
